# Hemelipoglycoprotein from the ornate sheep tick, *dermacentor marginatus*: structural and functional characterization

**DOI:** 10.1186/1756-3305-4-4

**Published:** 2011-01-07

**Authors:** Jarmila Dupejova, Jan Sterba, Marie Vancova, Libor Grubhoffer

**Affiliations:** 1 Faculty of Science, University of South Bohemia, Branisovska 31, CZ-37005 Ceske Budejovice, Czech Republic; 2 Institute of Parasitology, Biology Centre of the Academy of Sciences of the Czech Republic, Branisovska 31, CZ-37005 Ceske Budejovice, Czech Republic

## Abstract

**Background:**

Tick carrier proteins are able to bind, transport, and store host-blood heme, and thus they function also as antioxidants. Nevertheless, the role of carrier proteins in ticks is not fully understood. Some of them are found also in tick males which do not feed on hosts to such an extent such as females (there are differences in male feeding in different tick species) and thus they are not dealing with such an excess of heme; some of the carrier proteins were found in salivary glands where the processing of blood and thus release of heme does not occur. Besides, the carrier proteins bind relatively low amounts of heme (in one case only two molecules of heme per protein) compared to their sizes (above 200 kDa).

The main aim of this study is the biochemical characterization of a carrier protein from the ornate sheep tick *Dermacentor marginatus*, hemelipoglycoprotein, with emphasis on its size in native conditions, its glycosylation and identification of its modifying glycans, and examining its carbohydrate-binding specificity.

**Results:**

Hemelipoglycoprotein from *D. marginatus *plasma was purified in native state by immunoprecipitation and denatured using electroelution from SDS-PAGE separated plasma. The protein (290 kDa) contains two subunits with molecular weights 100 and 95 kDa. It is glycosylated by high-mannose and complex *N*-glycans HexNAc_2_Hex_9_, HexNAc_2_Hex_10_, HexNAc_4_Hex_7_, and HexNAc_4_Hex_8_. The purified protein is able to agglutinate red blood cells and has galactose- and mannose-binding specificity. The protein is recognized by antibodies directed against plasma proteins with hemagglutination activity and against fibrinogen-related lectin Dorin M from the tick *Ornithodoros moubata*.

It forms high-molecular weight complexes with putative fibrinogen-related proteins and other unknown proteins under native conditions in tick plasma. Feeding does not increase its amounts in male plasma. The hemelipoglycoprotein was detected also in hemocytes, salivary glands, and gut. In salivary glands, the protein was present in both glycosylated and nonglycosylated forms.

**Conclusion:**

A 290 kDa hemelipoglycoprotein from the tick *Dermacentor marginatus*, was characterized. The protein has two subunits with 95 and 100 kDa, and bears high-mannose and complex *N*-linked glycans. In hemolymph, it is present in complexes with putative fibrinogen-related proteins. This, together with its carbohydrate-binding activity, suggests its possible involvement in tick innate immunity. In fed female salivary glands, it was found also in a form corresponding to the deglycosylated protein.

## Background

Ticks are obligate ectoparasites of mammals, birds, and reptiles which feed only on blood of their hosts. Blood provides a rich source of nutrients needed for processes associated with development to the next life-stage and egg production. During the digestion of the blood, erythrocytes are lysed and heme is released. As ticks do not possess the ability to synthesize heme [1], they utilize the host-blood heme. For this reason, as well as to neutralize the toxic effects of free heme, ticks use carrier proteins which bind and store it. Their nomenclature is not uniform and the proteins are called different names; some of them are described below.

Hemelipoglyco-carrier proteins (CP) are the most abundant proteins in the hemolymph [2,3]. The best studied are the CPs in hard ticks, *Dermacentor variabilis *[4] and *Rhipicephalus microplus *[2]. Hemelipoprotein (HeLp), carrier protein isolated from *R. microplus*, has molecular weight of about 354 kDa and consists of two subunits with 103 kDa and 92 kDa. It occurs mainly in hemolymph of adult tick stages in concentrations of around 50 mg/ml and is one of the most abundant hemolymph proteins. This molecule is able to bind heme in the ratio of two moles of heme to one mole of native HeLp and contains 3% carbohydrates, and 33% lipids [2]. HeLp carbohydrates contain mainly mannose which comprises more than 90% of all carbohydrates present in HeLp which corresponds to glycans found in ticks [[5], unpublished results]. The protein contains also neutral lipids, phospholipids, cholesterol esters, and cholesterol oleate. Labeling of HeLp with ^55^Fe showed that this protein participates in heme transportation from hemolymph into ovaries during oogenesis [2].

Another CP, hemelipoglyco-carrier protein (DvCP), found in hemolymph of both male and female ticks *D. variabilis*, shows a significant sequence homology with HeLp [4-7]. DvCP has a molecular weight of 210 kDa and has two subunits with molecular weight of 98 kDa and 92 kDa. Similarly to HeLp, DvCP contains lipids and carbohydrates [4]. DvCP was localized to fat body, salivary glands, ovary, and muscles of partially fed females [3].

The role of carrier-proteins in ticks is not fully understood. Some of them are found also in males which engorge a more limited volume of blood and thus are dealing with lower amounts of heme [4]. Moreover, the carrier protein persisted in *D. variabilis *males hemolymph in higher concentrations and for a longer period of time after detachment as compared to tick females [4]. Thus, it is possible that these proteins play also roles other than transport and storage of the heme.

In the current study, hemelipoglycoprotein (HLGP) from the hemolymph of the tick *D. marginatus*, closely related to DvCP, was isolated and characterized by electrophoretic and blotting techniques, surface plasmon resonance (SPR) and MALDI-TOF/TOF (Matrix-assisted laser desorption/ionization-Time-of-flight/Time-of-flight) and ESI-FT-ICR (Electrospray ionization-Fourier-transform ion cyclotron resonance) mass spectrometry analysis. The molecular weight of the protein and its subunits, *N*-linked glycans modifying the protein, and carbohydrate-binding specificity were determined. The protein was also recognized by antibodies directed against Dorin M, a fibrinogen-related lectin from *Ornithodoros moubata *[8], and sera raised against hemagglutination activity of the *D. marginatus *hemolymph.

## Methods

### Ticks

Unfed and partially-fed (further referred to as "unfed" and "fed") females and males of the tick *Dermacentor marginatus *were obtained from the tick facility of the Institute of Parasitology, Biology Centre of the Academy of Sciences of the Czech Republic in České Budějovice. After metamorphosis, males and females were separated and kept in glass vials in wet chambers at 26°C until feeding/plasma and tissue preparation. Females and males were allowed to feed on laboratory guinea pigs for 6 days.

### Plasma and tissue preparation

Hemolymph was collected after cutting off a part of tick's anterior leg with fine scissors. The hemolymph from 8 to 10 ticks (approx. 10-15 μl and 5 μl for unfed and fed ticks, respectively) was collected directly into 50 μl of 0.9% NaCl containing protease inhibitors (Pierce, Thermo Fisher, Rockford, IL). The solution was centrifuged at 4°C for 10 min at 100 g to pellet the hemocytes. The resulting supernatant was then clarified at 23000 g for 20 min and both the plasma and hemocyte fractions were stored at -20°C.

Gut and salivary glands were dissected from partially-fed females, thoroughly washed in phosphate-buffered saline, pH 7.4 (PBS) to remove possible contamination, and organs from five ticks were homogenized with 300 μl PBS for 2 minutes at frequency of 30 Hz in TissueLyser II (Qiagen, Hombrechtikon, Switzerland) and stored at -70°C.

### SDS-PAGE and Blue Native/SDS-PAGE electrophoresis

For SDS-PAGE, plasma samples were diluted 1:5 in PBS, mixed with loading buffer with or without reducing agent dithiothreitol (Fermentas, Thermo Fisher, Vilnius, Lithuania) and heated for 5 min at 95°C. SDS-PAGE was performed on 4-17.5% gradient gels [9] in Mini-PROTEAN electrophoresis system (Bio-Rad, Hercules, CA). Gels were stained with PageBlue Protein Staining Solution (Fermentas). Approximately 5 μg proteins were loaded in the case of tick plasma and hemocytes, while 15 μg of tick gut and salivary gland proteins were applied.

In the case of BN-PAGE/SDS-PAGE, samples were mixed with 5% glycerol and 0.01% Ponceau S and separated on native gradient gel (3.5% stacking, 6-13% separating gel) [10] together with native protein molecular weight standards (Sigma-Aldrich, St. Louis, MO) in Mini-PROTEAN electrophoresis system. Gels were stained with PageBlue Protein Staining Solution or used for second dimension of 2 D electrophoresis. For the second dimension, the BN-PAGE separated proteins were used for Tris-Tricine SDS-PAGE [10]. Briefly, 12% separating gel was prepared, gel strips from BN-PAGE were incubated for 1 h in cathode buffer (0.1 M Tris, 0.1 M Tricine, 0.1% SDS, pH 8.25), inserted above the separating gel and overlaid with 4% stacking gel. Electrophoresis was performed for about 2.5 hours at a current limit of 50 mA using anode buffer (0.1 M Tris, 0.0225 M HCl, pH 8.9) and cathode buffer [10]. The separated proteins were stained with PageBlue Protein Staining Solution.

### Electroelution of HLGP

Electroelution was performed using ElutaTube™Protein, DNA and RNA Extraction and Dialysis Kit (Fermentas). Bands corresponding to HLGP were cut out of the gel, placed into ElutaTube vial with electrophoresis running buffer (0.025 M Tris, 0.192 M glycine, 0.1% SDS), and placed in the supporting tray in the electrophoresis tank with running buffer. Electroelution ran for 3 hours at 100 V. Samples were precipitated by four volumes of acetone, pelleted, the precipitated proteins were air-dried, and dissolved in water with resulting concentration of approximately 0.5 mg/μl.

### Anti-HLGP and anti-HA polyclonal serum preparation

Mice were housed in the Animal facility of the Institute of Parasitology, Biology Center of the ASCR in České Budějovice in plastic cages with sawdust bedding. Pellet diet and water were supplied *ad libitum *and mice were handled in accordance with the Animal Act of the Czech Parliament.

*D. marginatus *plasma proteins were separated by SDS-PAGE and stained with PageBlue Protein Staining Solution (Fermentas). HLGP bands (corresponding to approximately 50 μg of protein) were cut out and homogenized with 240 μl PBS (1×). Incomplete Freund's adjuvant was added in a 1:1 ratio and 80 μl of this solution was subcutaneously injected to BALB/c mice. Immunization was repeated 3× every 14 days by subcutaneous injection of 80 μl of this solution. Blood sera were collected 14 days after the last immunization. Sera were supplemented with glycerol (1:1), aliquoted and stored at -20°C.

Anti-hemagglutination activity (anti-HA) serum was prepared as described elsewhere [11]. 20 μl of tick hemolymph was added to 50 μl of 2% suspension of mouse red blood cells. After 1 h incubation, the hemagglutinated cells were washed, resuspended in 200 μl of PBS, and 50 μl of the mixture was injected into mice. The immunization was repeated three times every 14 days. Blood sera were collected 14 days after the last immunization. Sera were supplemented with glycerol (1:1), aliquoted and stored at -20°C.

### Immunoblotting

The electrophoretically separated proteins were transferred to PVDF membrane [12] for 1 hour at 20 V. The PVDF membrane was washed in PBS, cut into strips, and incubated for 1 hour in 5% skim powdered milk in PBS. Strips were then incubated for 1 hour in mouse anti-HLGP or anti-HA serum, washed with PBS-Tween 20 (0,05% Tween 20 in PBS) and incubated with goat anti-mouse antibody conjugated with alkaline phosphatase (Vector Laboratories, Burlingame, CA) in 5% milk. After incubation, strips were washed with PBS-Tween 20 and PBS. Reaction was developed in alkaline phosphatase-staining solution (Vector Laboratories) and after the development of sufficient signal it was stopped by washing the strips several times in Milli-Q water.

### Schiff staining and lectinoblotting of HLGP

Electroeluted HLGP was separated by SDS-PAGE and electroblotted to PVDF membrane. The presence of glycosylation was detected with DIG Glycan Detection Kit (Roche Applied Science, Mannheim, Germany) and the glycan types modifying the HLGP were identified using DIG Glycan Differentiation Kit (Roche Applied Science) utilizing lectins SNA, GNA, DSA, PNA, and MAA II, conjugated with digoxigenin.

Membrane strips for Schiff staining were washed with PBS, the glycoproteins were oxidized by 10 mM sodium periodate in 0.1 M sodium acetate buffer, pH 5.5 at room temperature for 20 minutes, washed in PBS, and incubated with DIG-3-O-succinyl-ε-aminocaproic acid hydrazide in sodium acetate buffer for 1 hour at room temperature. After three washes in Tris-buffered saline, pH 7.5 (TBS, 0.05 M Tris, 0.15 M NaCl) the strips were blocked in blocking solution (provided by the manufacturer and diluted 1:9 in TBS) for 1 hour at room temperature, washed in TBS, incubated with anti-DIG antibodies conjugated with alkaline phosphatase in the blocking solution for 1 hour at room temperature, washed with TBS, and finally, the color reaction was developed in BCIP/NBT solution in 0.1 M Tris, 0.05 M MgCl_2_, 0.1 M NaCl, pH 9.5. The reaction was stopped by thorough washing in Milli-Q water.

For lectinoblotting, the membrane strips were washed in TBS and blocked in blocking solution for 1 hour at room temperature. Next, the strips were washed in TBS and in TBS supplemented with 1 mM CaCl_2_, 1 mM MgCl_2_, 1 mM MnCl_2_, and incubated in the supplemented TBS with the individual DIG-conjugated lectins for minimum 1 hour at room temperature. The strips were washed again in TBS, incubated with anti-DIG antibodies conjugated with alkaline phosphatase in the blocking solution for 1 hour at room temperature, washed with TBS, and the color reaction was developed as described above.

### Enzymatic deglycosylation

Electroeluted HLGP was deglycosylated using glycosidases Endo H (New England Biolabs, Ipswich, MA) or PNGase F (New England Biolabs) and PNGase A (Roche Applied Science) under reducing and non-reducing conditions. For deglycosylation using PNGase A/F under reducing conditions, 68 μl of electroeluted HLGP was mixed with 10 μl denaturation buffer (5% SDS, 0.4 M DTT) and heated on 95°C for 10 min. The solution was mixed with 10 μl 0.5 M sodium phosphate, pH 7.4, 10 μl Nonident P-40, 0.5 μl PNGase A, 0.5 μl PNGase F, and 1 μl Milli-Q water.

For deglycosylation using Endo H under reducing conditions, 68 μl of electroeluted HLGP was mixed with 10 μl denaturation buffer (5% SDS, 0.4 M DTT) and heated on 95°C for 10 min. The solution was mixed with 10 μl 0.5 M sodium citrate, pH 5.5, 2 μl Endo H, and 10 μl Milli-Q water. Deglycosylation under non-reducing conditions was performed similarly with the denaturation step to be omitted. Deglycosylation reactions were performed overnight at 37°C. All reactions were performed in duplicates.

*N*-linked glycans from HLGP and *D. marginatus *hemolymph were prepared from reduced and alkylated electroeluted proteins, which were digested by trypsin (Roche Applied Science) in PBS, overnight at 37°C. The peptides were purified using C18 spin columns (Harvard Apparatus, Holliston, MA), vacuum-dried and deglycosylated as described above using a mixture of PNGase A and PNGase F to ensure the release of all *N*-glycans. The released glycans were purified using a combination of C18 and active-charcoal spin columns (Harvard Apparatus) [13]. *N*-glycans preparation and their subsequent MS analysis were performed in duplicates.

### Solid-phase permethylation of *N*-glycans for MS analysis

PNGase A/F-released glycans were vacuum dried, resuspended in 5 μl of water, and 70 μl of dimethylformamide and 25 μl of methyl iodide were added. The mixture was applied onto NaOH beads (Sigma-Aldrich) in spin-columns (Harvard Apparatus), incubated for 15 minutes, and the columns were centrifuged for 1 minute at 1500 × G. 25 μl of methyl iodide was added to the solution, again applied onto NaOH beads, incubated for 15 minutes, and centrifuged [13]. Next, the beads were washed with acetonitrile, centrifuged, and the solutions were pooled. The permethylated glycans were recovered by liquid/liquid extraction with chloroform and washing with 0.5 M NaCl and HPLC-grade water. Finally, the glycans were vacuum dried.

### Immunoprecipitation

50 μl of *D. marginatus *hemolymph plasma, diluted 1:5 in 0.9% NaCl, was mixed with 1 μl of 1% Tween 20 in PBS, 5 μl of protease inhibitors (Pierce), and with 444 μl of PBS. The mixture was incubated with 5 μl of anti-HA/anti-HLGP serum at 4°C overnight. 10 μl of magnetic Dynabeads with Protein G (Invitrogen, Carlsbad, CA) were added into the solution and incubated 45 min at room temperature. The beads were then washed six-times in 0.01% Tween 20 in PBS. HLGP was eluted using elution buffer containing primary amines (MicroLink Protein Coupling Kit, Pierce). Immunoprecipitation experiments were performed three-times with similar results.

### Surface plasmon resonance (SPR)

Quantitative measurement of interactions between HLGP and carbohydrates was performed on BiaCore 3000 instrument (GE Healthcare, Buckinghamshire, UK) (National Centre for Biomolecular Research, Michaela Wimmerová and Lenka Malinovská, Masaryk University, Brno, Czech Republic). A chip with immobilized monosaccharides (α-D-galactose on channel 1, α-D-mannose/α-L-fucose on channel 2, α-D-mannose on channel 3, and α-L-fucose on channel 4) was used. 15 μl of samples were injected on the chip and the response of individual channels was monitored at a flow-rate of 5 μl/min. Dilutions of the samples as well as samples containing EDTA (25 mM) were injected for complementary experiments. Both experiments with and without EDTA were performed in duplicates.

### Hemmaglutination assay

The determination of hemagglutination activity (HA) was performed in 96-well U-shaped microtitration plates by serial two-fold dilution of 50 μl samples in 50 μl of 0.15 M NaCl [9]. Next, 50 μl of 2% (v/v) suspension of mouse erythrocytes in 0.15 M NaCl was added to each well. The titer of HA was evaluated after 1 h incubation at room temperature and expressed as the reciprocal value of the last sample dilution causing visible agglutination.

### Mass spectrometry

Purified HLGP or gel slices containing HLGP were trypsinized according to the manufacturer's instructions (Roche Applied Science) and subjected to LC-MS analysis. Peptides were separated by HPLC on C18 silica (Agilent, Santa Clara, CA) using acetonitrile gradient (5-80%) as a mobile phase and analyzed by FT-ICR mass spectrometer (Thermo Fisher). The obtained data were compared to *Acari *non-redundant and Swiss-Prot databases using Mascot (a proprietary identification program, Matrix Science, Boston, MA) using strict criteria.

The permethylated glycans were spotted on matrix-assisted laser desorption/ionization (MALDI) plate with 2,5-dihydroxybenzoic acid and analyzed on 4800 MALDI TOF/TOF Analyzer (Applied Biosystems, Life Technologies, Carlsbad, CA) in positive-ion mode. The data were converted to mzXML format and further analyzed by mMass [14] and Glycoworkbench [15] software. Mass spectrometric analyses were performed in National Center for Glycomics and Glycoproteomics, Indiana University, Bloomington, IN, USA (Benjamin F. Mann, William R. Alley, Jr., and Milos V. Novotny).

## Results

### HLGP detection and identification in *D. marginatus *hemolymph

**Figure 1:**
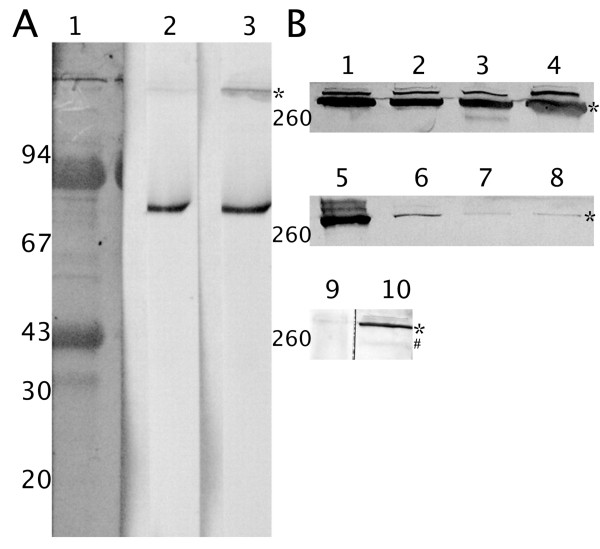
**Identification of putative FReD proteins in *D. marginatus *plasma and detection of HLGP in tick tissues**. Molecular weights of standard proteins are depicted. A) Immunoblotting of non-reduced electrophoretically separated *D. marginatus *plasma proteins. 1 - *D. marginatus *plasma proteins, stained with Coomassie Brilliant Blue, 2 - immunostaining of plasma proteins using antibodies against HA of *D. marginatus *hemolymph serum, 3 - immunostaining of plasma proteins by anti-Dorin M antibodies. B) Immunoblotting of HLGP in *D. marginatus *tissues using mouse polyclonal anti-HLGP serum. 1 - fed female plasma, 2 - unfed female plasma, 3 - fed male plasma, 4 - unfed male plasma, 5 - fed female hemocytes, 6 - unfed female hemocytes, 7 - fed male hemocytes, 8 - unfed male hemocytes, 9 - fed female gut, 10 - fed female salivary glands. * marks the position of native glycosylated HLGP, # marks the deglycosylated form of HLGP.

Putative lectin molecules in the hemolymph of the tick *Dermacentor marginatus *were detected using sera and antibodies recognizing similar molecules in tick species. The sera used were directed against HA of the hemolymph of *D. marginatus *tick in addition to antibodies recognizing Dorin M protein, a lectin from the hemolymph of the tick *Ornithodoros moubata*. Several putative carbohydrate-binding proteins with molecular weight around 37 kDa, 79 kDa, 80 kDa, and a high-molecular protein with molecular weight of approximately 290 kDa were identified in non-reduced hemolymph (Figure 1A). The reactivity of antibodies differed for these proteins and the 290 kDa molecule was the only one, recognized by all the antibodies.

The bands containing all four proteins were excised from the SDS-PAGE gel and mass spectrometric analyzes (ESI-FT-ICR MS) were performed. Although the three low molecular weight proteins were not identified, the 290 kDa protein (asterisk, Figure 1A) was identified as hemelipoglycoprotein (HLGP) related to hemelipoglyco-carrier protein (CP) from *D. variabilis*. The amino acid sequence coverage was 10.2% and 4.0% (see Additional File 1; Table S1 and Additional File 2; Table S2, respectively) for hemelipoglycoprotein precursor 1 and 2 of the homologous HLGP from *D. variabilis*, respectively.

Next, polyclonal antibodies against this protein were raised. The following immunoblot analysis showed that the anti-HLGP antibodies recognized the 290 kDa protein under non-reducing conditions and two subunits with molecular weights of 95 and 100 kDa under reducing conditions (data not shown).

### Isolation of HLGP

Further, HLGP was isolated from the plasma under both native and denaturing conditions. Native protein was purified using immunoprecipitation with Protein G coupled to magnetic beads and anti-HLGP serum (see Additional File 3; Figure S1; asterisk). The denatured protein was isolated from electrophoretically separated hemolymph by cutting out the HLGP protein from the gel and its subsequent electroelution (see Additional File 3; Figure S1; asterisk). In both cases, the purification of HLGP was successful.

### Characterization of HLGP

**Figure 2:**
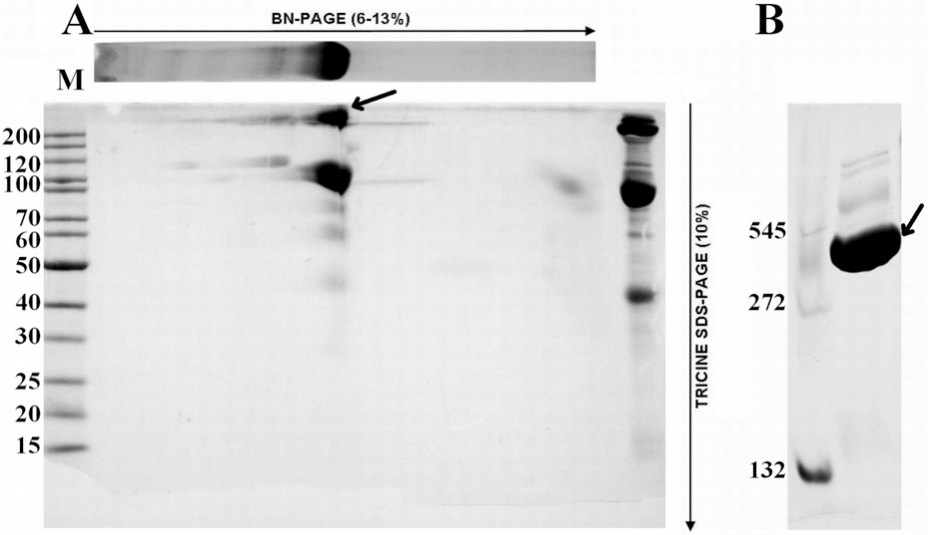
**2 D and native electrophoreses of plasma proteins and HLGP**. A) Blue native/SDS-PAGE 2 D electrophoretic separation of *D. marginatus *plasma proteins, stained by Coomassie Brilliant Blue (CBB). BN-PAGE separated plasma proteins are showed above the gel and acrylamide gradients are depicted above the gel (native electrophoresis) and on the right side (SDS-PAGE electrophoresis). HLGP is marked with an arrow. B) Blue native PAGE of *D. marginatus *plasma proteins, stained by CBB. 1 - native molecular weight standard, 2 - tick plasma. Complex of HLGP with other proteins is marked with an arrow.

To assess the molecular weight of the protein under native conditions, blue native-PAGE and subsequent SDS-PAGE of tick plasma were performed. The results show that HLGP (arrow, Figure 2A) is part of a high-molecular weight complex together with some other proteins which have not been identified yet (Figure 2A). Under native conditions, the complex has size of approximately 450 kDa (arrow, Figure 2B). However, some of these proteins are recognized by antibodies directed against tick lectins and HA (data not shown).

**Figure 3:**
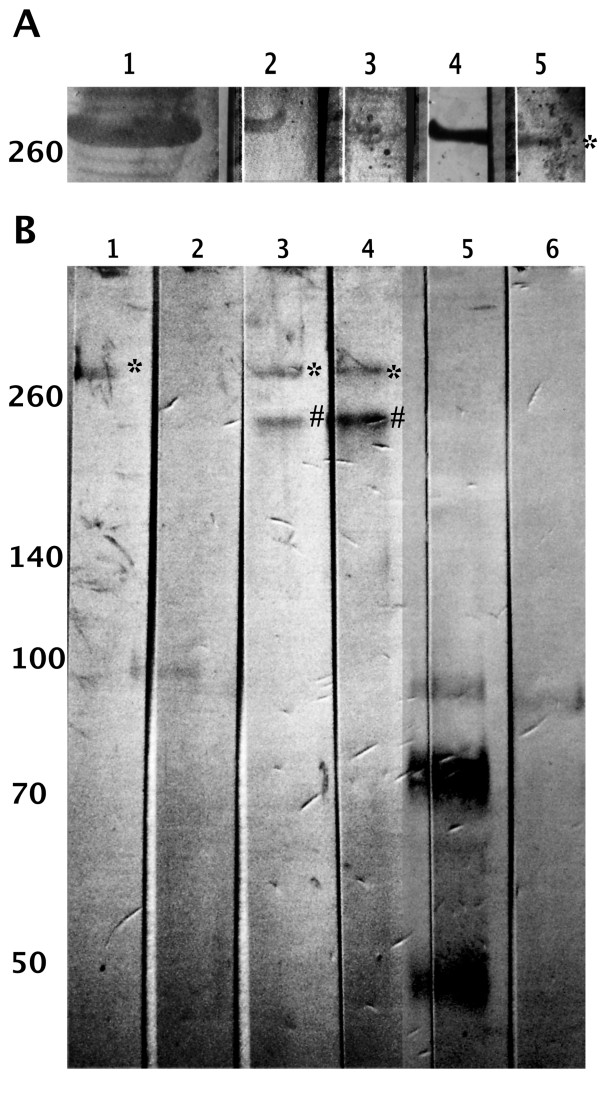
**HLGP is a glycosylated protein**. Molecular weights of standard proteins are depicted. A) Schiff staining and lectin staining of electrophoretically separated and electroblotted purified HLGP. 1 - tick plasma proteins, stained with Coomassie Brilliant Blue, 2-5 electroblotted HLGP staining. 2 - Schiff staining, 3 - SNA lectin staining, 4 - GNA lectin staining, 5 - DSA lectin staining of purified HLGP. Positive reaction in Schiff staining confirms the presence of glycans on HLGP. Types of glycans identified by positive reactions in lectinoblotting are described in text. B) Immunodetection of deglycosylated purified HLGP using anti-HLGP serum. 1,3,4 - non-reduced electroeluted HLGP and 2,5,6 - reduced electroeluted HLGP stained with anti-HLGP serum. Samples in lanes 3 and 5 were deglycosylated using Endo H, samples in lanes 4 and 6 deglycosylated using PNGase A/F. * - native glycosylated HLGP, # - deglycosylated form of HLGP.

Schiff staining of HLGP showed, that the protein is indeed glycosylated (Figure 3A, lane 2). The glycan moieties were further analyzed by lectin-affinity staining. Terminal mannose-specific GNA lectin and Gal-β(1-4)-GlcNAc-specific DSA lectin showed strong binding to HLGP (Figure 3A, lanes 4,5), α(2-6)-linked sialic acid-specific SNA lectin stained HLGP weakly (Figure 3A, lane 3), while Gal-β(1-3)-GalNAc and α(2-3)-linked sialic acid-specific lectins PNA and MAA did not give positive reaction (data not shown). These results suggest the presence of *N*-glycans of hybrid and complex type with terminal mannose, galactose or sialic acid. DSA lectin binding to HLGP can indicate the presence of *O*-glycans, too.

The proportion of the glycan part of the molecule was determined from deglycosylation reactions of HLGP. Enzymes Endo H and the mixture of PNGase A/F were used to cleave off the *N*- glycans. Anti-HLGP serum detected two proteins with 250 kDa and 290 kDa in both PNGase A/F (Figure 3B, lane 3) and Endo H treated purified native HLGP (Figure 3B, lane 4). As these endoglycosidases work under native conditions with a slower reaction rate, the larger protein (asterisks) presumably represents the glycosylated form and the 250 kDa protein (hashes) the deglycosylated form of the protein. Thus, the size of the *N*-glycan moieties was estimated to approximately 40 kDa.

**Figure 4:**
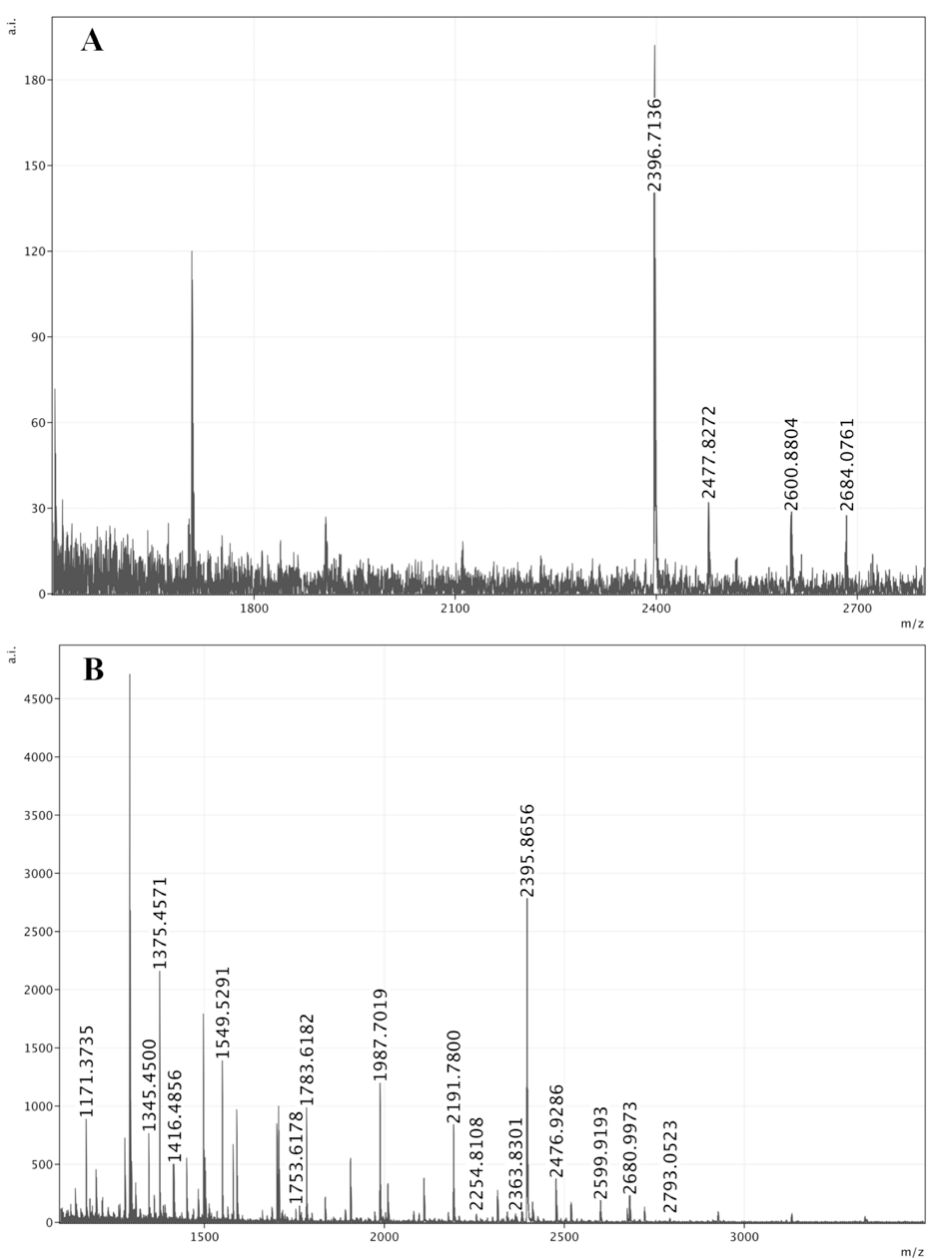
**Mass spectra of *N*-glycans from the purified HLGP and from *D. marginatus *hemolymph**. A) MALDI-TOF spectrum of enzymatically released *N*-glycans from HLGP. Four peaks corresponding to s HexNAc_2_Hex_9 _(m/z 2396.7), HexNAc_2_Hex_10 _(m/z 2600.8), HexNAc_4_Hex_7 _(m/z 2477.8), and HexNAc_4_Hex_8 _(m/z 2684) are marked. Three more peaks are visible corresponding to contaminating series of hexoses and *O*-glycans (not labeled). B) MALDI-TOF spectrum of enzymatically released *N*-glycans from *D. marginatus *hemolymph. A range of glycan structures was identified, namely high-mannose type glycans HexNAc_2_Hex_3 _to HexNAc_2_Hex_10 _(m/z 1171.3, 1375.4, 1783.6, 1987.7, 2191.8, 2395.9, 2599.9) and complex fucosylated and non-fucosylated glycans such as HexNac_3_Hex_3 _(m/z 1416.5), HexNAc_2_Hex_4_dHex_1 _(m/z 1549.5), HexNAc_2_Hex_5_dHex_1 _(m/z 1753.6). Again, contaminants were present in the sample - series of hexoses as well as *O*-glycans (not labeled).

MALDI-TOF/TOF analysis of PNGase A/F released, permethylated *N*-glycans from purified HLGP provided the information on their masses and composition. The MS spectra showed presence of high-mannose structures HexNAc_2_Hex_9 _and HexNAc_2_Hex_10 _(ion m/z 2396.7 and 2600.8, respectively) and complex glycans HexNAc_4_Hex_7 _and HexNAc_4_Hex_8 _(m/z 2477.8 and 2684, respectively) (Figure 4A). Composition of the glycan structures was confirmed by MS/MS analysis (data not shown). Spectra contained also contaminating series of hexose ions and of *O*-glycans (e.g. ion m/z 1707, not marked in the figure), present also in other tick samples such as salivary glands, gut, hemolymph, ovaria, or whole tick homogenates (unpublished results). For comparison, *N*-glycans from *D. marginatus *hemolymph were also analyzed. All four glycan structures, identified in HLGP samples, were present also in hemolymph (Figure 4B). Other hemolymph protein *N*-glycans were identified as high-mannose type ranging from HexNAc_2_Hex_3 _to HexNAc_2_Hex_8 _(m/z 1171.3, 1375.4, 1783.6, 1987.7, 2191.8) and fucosylated and non-fucosylated complex type glycans such as HexNac_3_Hex_3 _(1416.5), HexNAc_2_Hex_4_dHex_1 _(1549.5), HexNAc_2_Hex_5_dHex_1 _(1753.6). Also in this case, a series of hexoses as well as *O*-glycans were present in the sample (Figure 4B).

**Figure 5:**
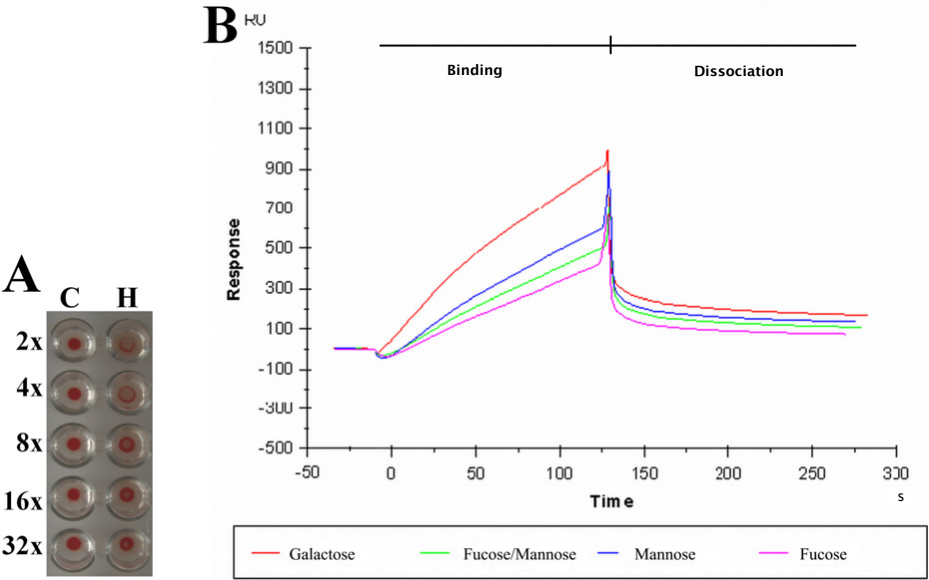
**HLGP is a carbohydrate-binding protein - HA and SPR**. A) Hemagglutination activity of purified HLGP. C - negative control (no hemagglutination activity), H - serial two-fold dilution of electroeluted HLGP. Hemagglutination activity is visible in dilutions 2× to 16×. B) Carbohydrate-binding specificity of purified HLGP assessed by SPR on a chip with four channels arranged sequentially as galactose-fucose/mannose-mannose-fucose. Red line corresponds to signal from the channel with immobilized galactose, green to channel with immobilized fucose and mannose, blue to channel with immobilized mannose and purple to channel with immobilized fucose. The fucose/mannose channel contained half amount of the corresponding carbohydrates and serves as control. Lines correspond to SPR response in real-time (not normalized for the sequential flow through channels). Galactose showed the strongest binding by HLGP, some binding was observed also for mannose. Fucose was not recognized by the protein.

In accordance with the presence of hemagglutination activity (HA) in hemolymph (unpublished results), the purified HLGP demonstrated HA (Figure 5A). The inhibition of HA with monosaccharides was not performed due to low amounts of sample available and thus the carbohydrate specificity was addressed by surface plasmon resonance experiments. A chip with channels with immobilized galactose, fucose, and mannose was used. The channel with immobilized fucose and mannose (half amounts) was used as a control of binding-response. The experiment showed binding specificity of the purified HLGP towards galactose (Figure 5B). HLGP showed also a weak binding-specificity towards mannose as seen from comparison of the signal from the mannose channel and the control fucose/mannose channel (Figure 5B). The binding of HLGP to carbohydrates was inhibited by EDTA (data not shown).

### Tissue distribution

The presence of HLGP in tick tissues was determined by immunoblotting. In addition to plasma, hemocytes (as the cellular component of hemolymph) from both fed and unfed females and males, fed female salivary glands (to investigate the presence of HLGP in salivary glands and possibly also in saliva), and gut were investigated.

In all samples, anti-HLGP serum detected the same protein with the same molecular weight (Figure 1B). While amount of the protein was similar in both females and males, regardless of blood-feeding, HGLP was present in higher amounts only in hemocytes from fed female tick. In males, the amount of HLGP did not increase during the blood-feeding.

Interestingly, in the salivary glands (Figure 1B, lane 10), the anti-HLGP serum detected two forms of this protein, corresponding in size to the glycosylated (asterisk) and the deglycosylated (hash) forms (compare with Figure 3B, lanes 3,4).

## Discussion

This work was aimed towards the characterization of hemelipoglycoprotein, the most abundant protein of tick hemolymph. To date, only one function of the protein has been identified - the binding and storage of heme [2,4]. Surprisingly, in our work, the protein was recognized by antibodies, which were directed against the first isolated tick lectin Dorin M [8] but also sera against the hemagglutination activity of the hemolymph of ticks *D. marginatus *and *I. ricinus*. Hemagglutination activity (HA) is an ability of some proteins to bind carbohydrates on red blood cells and thus to form clusters. HA is a typical characteristic of lectins, proteins with the ability of specific recognition of specific carbohydrates and their reversible binding. Antibodies raised against HA of tick hemolymph recognize proteins which bind red blood cells in this manner and potentially also proteins, which form tight complexes with them. Anti-Dorin M antibodies, on the other hand, react more specifically with proteins containing FReD domain or lectins (unpublished results).

All the used antibodies detected similar proteins in hemolymph; one of the putative lectin molecules with the molecular weight of 290 kDa (with two subunits of 86 and 90 kDa) was identified as hemelipoglycoprotein (HLGP). The theoretical masses of the subunits for the HLGP from *D. variabilis*, based on convertase cleavage, are 85 and 93 kDa for HLGP precursor (GenBank accession No. EEC1882) and 79 and 92 kDa for HLGP precursor 2 (GenBank accession No. EEC17915) [16] which clearly corresponds with our results for *D. marginatus *protein. The molecular weight of the protein is similar to other tick hemelipoglycoproteins or carrier proteins (another name of the protein) whose size ranges from 200 kDa to 365 kDa [2,4]. The proteins, described by these authors, contained two subunits of 92 and 98 or 92 and 103 kDa.

Hemelipoglycoproteins contain the N-terminal lipoprotein domain, a domain of unknown function, and the von Willebrand domain type D, but they do not contain the fibrinogen-related domain, nor any other known lectin domain; moreover, hemelipoglycoproteins are not phylogenetically related to FReD proteins [17]. On the other hand, the N-terminal lipoprotein domain is proposed to have carbohydrate, lipid, and metal-binding properties [16]. The N-terminal lipoprotein domain shares high similarity with vitellogenins from insects and other arthropods and the carbohydrate-binding specificity of vitellogenin was showed for example in the Colorado beetle hemolymph (*Leptinotarsa decemlineata*) [18]. The lipoprotein domain of the DvCP from *D. variabilis *is similar also to vitellogenin from fish of the genus *Branchiostoma*, which also shows hemagglutination activity and carbohydrate-binding ability [19].

HLGP and the other proteins recognized by the anti-HA antibodies in the *D. marginatus *hemolymph form a high-molecular weight complex under native conditions (Figure 2A, B). Thus, in the process of anti-HA antibodies preparation, HLGP could be co-purified with proteins with lectin activity. This can explain the anti-HA antibodies binding to HLGP; however, this fact does not explain the binding of anti-Dorin M antibodies to the protein. The structure of HLGP, FReD-containing proteins, and Dorin M are not known and HLGP does not show any similarity to FreD proteins on the primary structure level. However, recognition of HLGP by anti-HA and anti-Dorin M antibodies, together with carbohydrate-binding SPR studies suggest, that HLGP shares similar epitopes with the lectin domains of the FReD-containing proteins and that HLGP is structurally, rather than sequentially, similar to lectins/FReD-containing proteins.

The electroeluted and native purified HLGP were able to bind to immobilized monosaccharides in SPR experiments or to hemagglutinate red blood cells (Figure 5A, B). This corresponds to hemagglutination experiments performed on hemolymph from *D. marginatus *(unpublished results) as well as from other ticks [8,17,20]. The diminished carbohydrate-binding activity in the presence of EDTA (data not shown) suggests dependency on calcium (divalent) cations, which corresponds to calcium dependence of HA of crayfish plasma vitellogenin-related protein [21] and sea urchin vitellogenin [22].

FReD-containing proteins are present in vertebrates and in invertebrates. They were described as humoral factors of innate immunity which are able to recognize PAMPs (pathogen-associated molecular patterns); their other functions include participation in regulation of embryo development or in cell adhesion. Donohue and colleagues [16] and Maya-Monteiro and colleagues [2] suggest also other possible roles of carrier proteins in the ticks apart from the heme-storage and prevention of oxidative stress. Our finding of HLGP participation in a high-molecular weight complex with other putative FReD-containing proteins, the presence of two forms of HLGP in tick salivary glands, and its carbohydrate-binding properties point to some other, yet undiscovered, roles of the protein.

HLGP from *D. marginatus *is modified by *N*-glycans as was showed by Schiff staining and lectin affinity staining (Figure 3A). PNGase A/F and Endo H endoglycosidases decreased the size of HLGP by 40 kDa under native conditions (Figure 3B). The reactivity of HLGP with lectins DSA, GNA, and SNA indicated the presence of *N*-glycans with terminal mannose, terminal α(1-4)-linked galactose and α(2-6)-linked sialic acid. On the other hand, the absence of binding by the MAA II and PNA lectins suggested the absence of α(2-3)-linked sialic acid and of terminal α(1-3)-linked galactose. The subsequent mass spectrometric analysis of permethylated *N*-glycans from the purified HLGP revealed the presence of four different glycan moieties. Two were high-mannose type containing two *N*-acetylhexosamines and nine and ten hexose molecules, and the other two were complex glycans comprising four HexNAcs and seven and eight hexoses, possibly with terminal galactoses. Thus, the mass spectrometric analysis confirmed the results from lectin-affinity staining. Similar glycans were found also in other tick glycoprotein with identified glycans, Dorin M from *Ornithodoros moubata *[5]. In Dorin M, two high-mannose glycans and a core-fucosylated structure were identified.

Surprisingly, core-fucosylated *N*-glycans were not found in HLGP, even though their presence was confirmed in *D. marginatus *hemolymph (Figure 4B) and they are the most abundant type of glycans found in tick saliva and salivary glands (*Ixodes ricinus*, unpublished results). The role of core-fucosylation and protein-specific fucosylation in ticks will be one of the exciting problems of tick glycobiology and physiology.

HLGP has been detected in several tissues of the tick - hemolymph, hemocytes, gut, and the protein in both glycosylated and non-glycosylated form also in salivary glands. Previously, HLGP was immunolocalized inside the gut cells, on their surface, as well as inside the salivary duct and epithelium cells of salivary glands (unpublished results). Its presence was shown in these tissues also in other ticks and in different life stages of ticks [3,4]. Furthermore, HLGP is present in salivary glands of *D. marginatus *in two forms, one of them corresponding in size to the non-glycosylated form of the hemolymph (glycosylated) protein, the other to the hemolymph protein. While Gudderra and colleagues [3] showed, that DvCP in the salivary glands of *D. variabilis *does not contain heme in its molecule, the presence of two forms of the protein was not described to date elsewhere. The role of HLGP in tick saliva is not clear nor is the importance of the glycosylation of HLGP or the other tick salivary proteins for the tick feeding.

## Conclusion

A heme-carrier protein from the hemolymph of the ornate sheep tick *Dermacentor marginatus*, hemelipoglycoprotein, was characterized. The protein is *N*-glycosylated, bearing high-mannose and complex type glycans without core-fucose. Nevertheless, core-fucosylated glycans are abundant in hemolymph. Hemelipoglycoprotein forms a complex with fibrinogen-related proteins in hemolymph. It shows carbohydrate-binding activity, which is divalent cations-dependent. Moreover, the protein is present in two forms in fed female salivary glands and one of these forms corresponds to the deglycosylated protein. Altogether, these findings suggest also other roles of the protein in ticks different from its heme-binding function. Due to the carbohydrate-binding activity, one of the possibilities is the involvement of hemelipoglycoprotein in innate immunity processes.

## Abbreviations

AP: alkaline phosphatase; CP: carrier proteins; dHex: deoxyhexose; DSA: *Datura stramonium *agglutinin; FReD: fibrinogen-related domain; FReP: fibrinogen-related protein; GNA: *Galanthus nivalis *agglutinin; HA: hemagglutination activity; Hex: hexose; HexNAc: N-acetyl hexosamine; HLGP: hemelipoglycoprotein; MAA: *Maackia amurensis *agglutinin; PAMP: pathogen-associated molecular pattern; SNA: *Sambucus nigr*a agglutinin; SPR: surface plasmon resonance.

## Competing interests

The authors declare that they have no competing interests.

## Authors' contributions

JD conducted electrophoreses, blotting experiments, HA assay, and participated in SPR experiments, JD and JS participated in anti-HLGP and anti-tick HA sera preparation and in HLGP purification, JS and MV performed mass spectrometry and analysis of data, MV performed tick tissue preparation and participated in their analysis, LG co-ordinated the experiments, all authors participated in the design of experiments, manuscript preparation, and they approved the final manuscript.

## Supplementary Material

Additional File 1 Tryptic fragments identified in hemelipoglycoprotein precursor [*Dermacentor variabilis*], coverage 10.2%. Mass:178646, Score:12286, Queries matched: 244. Click here for file

Additional File 2 Tryptic fragments identified in hemelipoglycoprotein precursor 2 [*Dermacentor variabilis*], coverage 4%. Mass:171972, Score: 707, Queries matched: 12. Click here for file

Additional File 3 **Purification of HLGP**. Molecular weights of standard proteins are depicted. A) Immunoprecipitation of HLGP from *D. marginatus *hemolymph. 1 - Immunostaining of non-reduced HLGP in tick plasma, 2,3 - immunostaining of immunoprecipitated non-reduced HLGP. B) Electroelution of electrophoretically separated HLGP from *D. marginatus *plasma. SDS-PAGE separated proteins stained with Coomassie Brilliant Blue. 1 - non-reduced plasma proteins, 2 - non-reduced electroeluted HLGP. * marks the position of native glycosylated HLGP, # marks the deglycosylated form of HLGP. Click here for file
