# Supplementary figures and images for: Hemelipoglycoprotein from the ornate sheep tick, dermacentor marginatus: structural and functional characterization
Source: Parasit Vectors. 2011 Jan 7;4:4. doi: 10.1186/1756-3305-4-4 (PMC3022847; doi:10.1186/1756-3305-4-4)

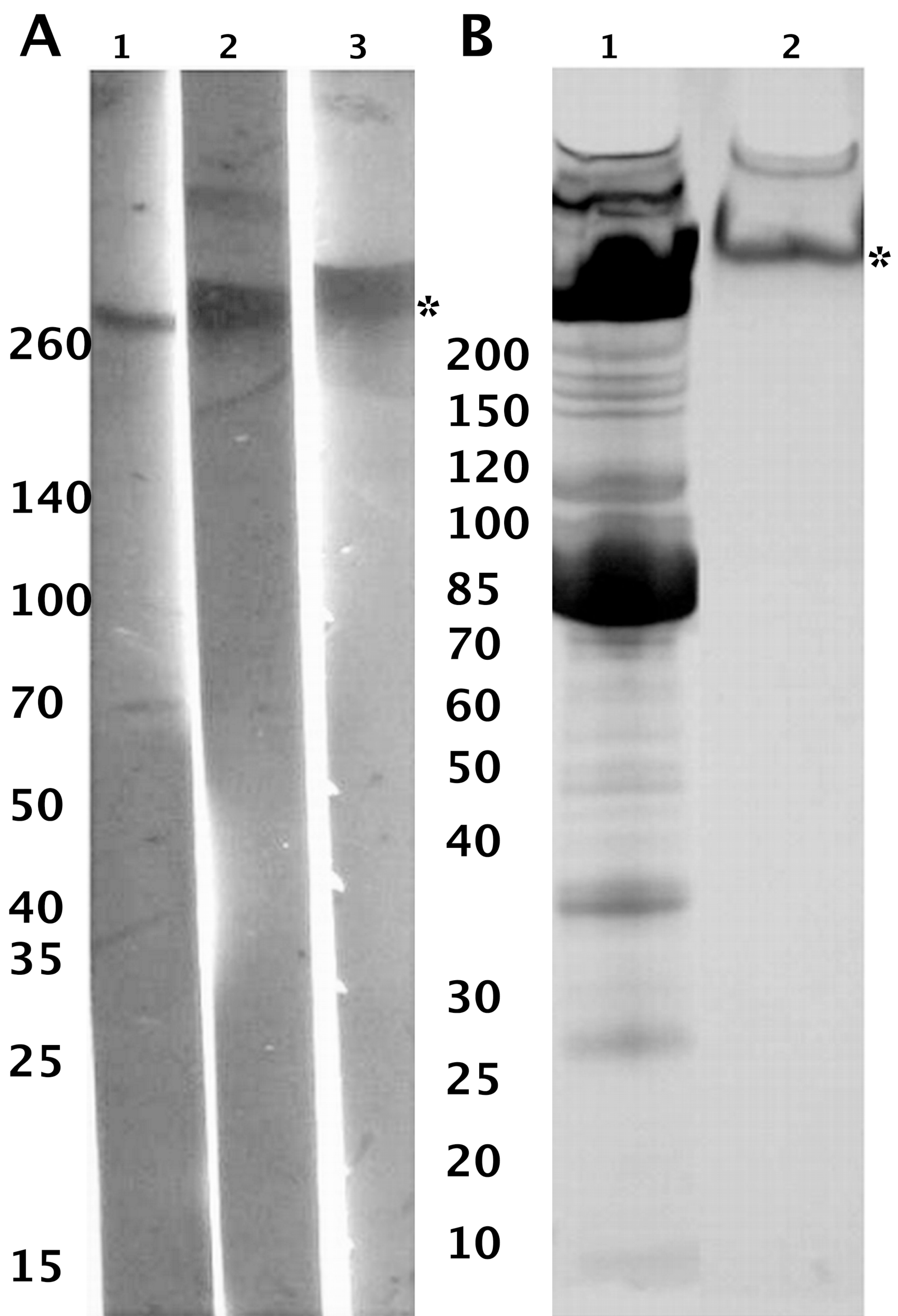

Supplement: Additional File 3 — Purification of HLGP. Molecular weights of standard proteins are depicted. A) Immunoprecipitation of HLGP from D. marginatus hemolymph. 1 - Immunostaining of non-reduced HLGP in tick plasma, 2,3 - immunostaining of immunoprecipitated non-reduced HLGP. B) Electroelution of electrophoretically separated HLGP from D. marginatus plasma. SDS-PAGE separated proteins stained with Coomassie Brilliant Blue. 1 - non-reduced plasma proteins, 2 - non-reduced electroeluted HLGP. * marks the position of native glycosylated HLGP, # marks the deglycosylated form of HLGP. [file 1756-3305-4-4-S3.PDF]
